# Food-Related Symptoms and Food Allergy in Swedish Children from Early Life to Adolescence

**DOI:** 10.1371/journal.pone.0166347

**Published:** 2016-11-15

**Authors:** Jennifer L. P. Protudjer, Mirja Vetander, Inger Kull, Gunilla Hedlin, Marianne van Hage, Magnus Wickman, Anna Bergström

**Affiliations:** 1 Institute of Environmental Medicine and Centre for Allergy Research, Karolinska Institutet, Stockholm, Sweden; 2 Centre for Allergy Research, Karolinska Institutet, Stockholm, Sweden; 3 Sachs’ Children and Youth Hospital, Södersjukhuset, Stockholm, Sweden; 4 Department of Clinical Science and Education and Centre for Allergy Research, Karolinska Institutet, Stockholm, Sweden; 5 Department of Women’s and Children’s Health and Centre for Allergy Research, Karolinska Institutet, Stockholm, Sweden; 6 Department of Medicine Solna, Immunology and Allergy Unit, Karolinska Institutet, and Karolinska University Hospital, Stockholm, Sweden; University of Kansas Medical Center, UNITED STATES

## Abstract

**Background:**

Risk factors for persistence of food-related symptoms (FRS) and food allergy (FA) from early life to adolescence are incompletely understood. The aim of this study was to identify risk factors for FRS and FA in adolescence amongst children with FRS or FA in the first four years of life (early life).

**Methods:**

In children enrolled in a Swedish birth cohort and followed to 16 years (n = 2572), we defined children with early life FRS in the absence of FA, and FA. Corresponding phenotypes were defined at 16 years. Associations between potential risk factors at 4 years and FRS and FA at 16 years were investigated using logistic regression.

**Results:**

Early life FRS and FA prevalences were 12.2% and 6.8%, respectively. Amongst children with early life FRS, 35.7% had FRS or FA at 16 years, whereas 74.3% of the children with early life FA had FA at 16 years. For each of the early life phenotypes, parental allergy, early life allergic multimorbidity, early life reactions to peanuts/tree nuts and IgE reactivity at 4 years were statistically significantly associated with FRS or FA at 16 years. In contrast, male sex was associated with an increased risk of FA at 16 years among children with early life FA only.

**Conclusions:**

In early life, food-related symptoms are twice as common as food allergy. Unlike food allergy, food-related symptoms often remit by adolescence. Yet, these phenotypes have many common risk factors for persistence to adolescence.

## Introduction

Adverse reactions to foods are common amongst children [1]. Food-related symptoms (FRS) that are not clinically diagnosed as allergy affect more children [2–4] than food allergy (FA) [5–7], but FA reactions tend to be more severe [1, 8]. The most severe reaction, anaphylaxis, has a peak incidence in early life [9, 10] and is potentially [8], but rarely [11] fatal. The prevalence rates of paediatric FRS [12] and FA [13] appear to be rising. Although many children outgrow reactions to food [14] including FA [5, 15] by school age, some children experience persistence through adolescence [2, 7]. Amongst adolescents, FA, but also FRS without known background mechanisms, are associated with poorer health-related quality of life compared to healthy controls [16, 17]. Yet, health-related quality of life does not appear to differ between the phenotypes [18]. Moreover, both phenotypes burden healthcare systems [19, 20], society [21] and households [21, 22].

Risk factors for early life FRS and FA have been studied. Family history of allergy [23–25] and allergic diseases in early life, particularly eczema [23] and Immunoglobulin E (IgE) reactivity [24–27], are established risk factors, whereas early life environmental factors and socio-demographic exposures remain incompletely understood [23, 25–30]. Less is known about the risk factors for, and the prognosis of FRS and FA from early life through adolescence. Therefore, we aimed to identify risk factors for FRS and FA in adolescence amongst children with FRS or FA in the first four years of life (early life).

## Methods

### Study design and population

This study is based on data from the BAMSE project [30], a longitudinal, population-based birth cohort of 4089 children born in Stockholm, Sweden between 1994 and 1996. Parents completed questionnaires at baseline (children 2–3 months old), and 1, 2, 4, 8, 12 and 16 years old. The response rate through 16 years was 78% (3181/4089) from baseline.

Children with information on parent-reported FRS and doctor-diagnosed FA at 1, 2, 4 years, and 16 years were included in the present study (n = 2572, 62.9% of the entire cohort). Information on allergen-specific IgE reactivity in serum was available in a subpopulation of participants at 4 years and at 16 years (n = 1903 and n = 2057, respectively), including 1617 participants for whom IgE information was available at both 4 and 16 years.

Ethical permission was obtained by the Regional Ethical Review Board, Karolinska Institutet, Stockholm, Sweden for each of the assessments: baseline, 1 year, 2 years (DNR 93:189); 4 years (DNR 98–175; 01–478), and; 16 years (DNR 2010/1474-31/3). Written informed consent was obtained from parents/guardians on behalf of their children.

### Definitions of FRS, FA, systemic reactions, anaphylaxis and markers of severity

#### Food-related symptoms (FRS)

Early life FRS: Children were classified as having early life FRS if their parent(s) reported a specific symptom(s) to a specific food(s) in the 12 months prior to the 1, 2 and/or 4- year questionnaires [31], but not doctor-diagnosed FA at any of these time points. Specific symptoms included vomiting, diarrhoea, eczema, urticaria, itch or swelling of the lips and/or eyelids, runny nose, and/or asthma. Specific foods included milk, egg, fish, tree nuts, peanuts, peas, soy, wheat, fruit with pits/pips, and at 4 years only, citrus, chocolate and banana. Foods reported to cause a reaction were labelled culprit foods.

FRS at 16 years: Similar to the definition in early life, this variable was based on parent-reported specific symptom to a specific food in the 12 months prior to the 16 year assessment, without doctor-diagnosed FA. Children were classified as having FRS if they continued to avoid a specific food due to a previous reaction(s) and/or due to results of previous allergy testing and did not have a doctor’s diagnosis of FA.

#### Food allergy (FA)

Early life FA: Children were classified as having early life FA if they had early life FRS (per the definition above) and parent-reported doctor-diagnosed food allergy at 1, 2 and/or 4 years.

FA at 16 years: This variable was based on FRS at 16 years in combination with parent-reported doctor-diagnosed food allergy at any age to 16 years.

#### FRS or FA at 16 years

This variable was based on FRS at 16 years or FA at 16 years and is the outcome used when assessing risks at this age in relation to early life FRS.

#### Systemic reaction at 16 years

This variable describes parent-reported reactions at 16 years involving reactions which cannot be considered localised symptoms: dermatological, respiratory or cardiovascular. It was defined for both FRS and FA at 16 years.

#### Anaphylaxis at 16 years

This variable is based on parent-reported data at 16 years corresponding to the currently accepted definition of anaphylaxis [32]; see S1 Table. Briefly, anaphylaxis was considered as any reaction involving at least two organ systems (gastrointestinal, dermatological, lower respiratory, cardiovascular) with specific, predefined symptoms. This variable was defined for both FRS and FA at 16 years [32].

#### Markers of severity of FRS and FA

Age at first reaction: Amongst those with early life FRS or FA, this variable was defined as first reaction by 2 years, or between 2–4 years.

Symptoms: Parent-reported symptoms upon ingestion of food and/or drink, were obtained from the 4 year questionnaire. (Data on symptoms were not collected as the 1- or 2 year questionnaires.) Symptoms were collapsed into three categories: gastrointestinal (vomiting and/or diarrhoea), dermatologic (urticaria and/or oedema) and respiratory (rhinitis and/or wheezing). Children were classified into the most severe category of symptoms reported by their parents.

Specific foods: Parent-reported reactions to individual foods were obtained from the 1, 2 and 4 year questionnaires. Presented herein are the seven most common culprit foods (milk, egg, fish, peanuts/tree nuts, fruits with pips or pits, citrus fruit and chocolate) in our study population. These categories of foods were non-mutually exclusive.

### Definitions of baseline characteristics, infant feeding, early life allergic multimorbidity and IgE reactivity

Definitions of baseline characteristics, infant feeding, early life allergy-related diseases (asthma, eczema and/or rhinitis) and IgE reactivity are shown in S2 Table.

#### IgE reactivity at 4 years and 16 years

Sera obtained at 4 years and at 16 years were analysed for IgE reactivity to common aeroallergens with Phadiatop® (cat, dog, horse, house dust mite, timothy, birth, mugwort, mould) and food allergens with fx5® (egg, milk, codfish, wheat, peanut, soy) (Thermo Fisher/Phadia AB, Uppsala, Sweden). Children were considered to have IgE reactivity if they had an IgE antibody level ≥0.35 kU_A_/l for Phadiatop^®^ and/or fx5^®^. Amongst children with early life FRS, information on IgE reactivity was available in a subset 71.7% (225/314) of children at 4 years and 79.0% (248/314) of children at 16 years. The corresponding numbers for FA were 73.7% (129/175) and 81.7% (143/175). Amongst these same groups of children, 13.7% (34/248) of children with early life FRS had IgE reactivity to foods at 16 years. The corresponding number for children with early life FA was 68/143 (47.6%).

Corresponding phenotypes were also created for FA. Food allergen-associated FA at 16 years was present in 38.9% (68/175) of children with early life FA.

### Statistical Analyses

Descriptive statistics included sample sizes, percentages, and 95^th^ per cent confidence intervals (95%CI). Proportional Venn diagrams were generated to illustrate the progression of FRS, from early life to 16 years.

Associations between exposures (baseline characteristics, infant feeding, early life allergic multimorbidity, markers of severity, IgE reactivity) and outcomes (FRS or FA at 16 years, FA at 16 years) were estimated using binary and multinomial logistic regression, and for which we reported the odds ratios (OR) and 95%CI.

The final models were adjusted for covariates selected based on *a priori* knowledge and included sex, parental allergy and socio-economic status. In a sensitivity analysis, we additionally included the variable ‘early life allergic multimorbidity’. This additional adjustment insubstantially altered (<10%) most point estimates, and did not statistically significantly alter any of the point estimates. As such, the results are not presented herein.

Analysis was performed with STATA Statistical Software (release13.1; StataCorp, College Station, Texas, USA).

## Results

Our study population (n = 2572) was comparable to the entire cohort with respect to participant characteristics, with the exception of small, but statistically significantly higher rates of white collar families (84.7% vs. 82.7%, respectively) and lower rates of tobacco smoking in pregnancy (11.4% vs. 12.9%, respectively) (S3 Table).

Children with early life FRS or FA were comparable to children without reactions or allergy to food regarding distribution of sex, parental migration, socio-economic status or infant feeding (Table 1). Early life asthma, eczema and rhinitis were all more common amongst children with early life FRS (63.4% had at least one of the diseases) and most common amongst children with early life FA (80.0%), compared to children with no early life reactions or allergy to food (38.4%).

**Table 1:** Distribution of baseline characteristics and parent-reported symptoms in early life in relation to early life food-related symptoms (FRS) and food allergy (FA).

	No early life reactions or allergy to food (N = 2083)			Early life FRS but not FA (N = 314)			Early life FA (N = 175)		
Baseline characteristics	n	Percent	95% CI	n	Percent	95% CI	n	Percent	95% CI
Females	1055	50.7	48.5–52.8	165	52.6	46.9–58.1	74	42.3	34.9–50.0
Swedish-born parents	1623	81.1	79.3–82.8	239	77.6	72.5–82.1	128	74.0	65.9–79.6
White collar families	1748	84.9	83.3–86.5	266	85.0	80.5–88.8	141	81.5	74.9–87.0
Parental allergy*	594	28.8	26.8–30.8	92	29.7	24.6–35.1	79	45.7	38.1–53.4
Infant feeding									
Exclusive breastfeeding for ≥ 4 months	1689	81.2	79.4–82.8	244	77.7	72.7–82.2	145	82.9	76.4–88.1
Early life allergic multimorbidity (to 4 years)									
Asthma	210	10.0	8.9–11.5	49	15.7	11.9–20.2	45	26.6	20.2–33.8
Eczema	529	25.4	23.6–27.4	150	48.1	42.4–53.8	127	72.6	65.3–79.0
Rhinitis	199	9.8	8.5–11.2	70	22.7	18.1–27.7	74	43.0	35.5–50.8
At least one of the above	799	38.4	36.3–40.5	199	63.4	57.8–68.7	140	80.0	73.3–85.7

*Doctor-diagnosed asthma *and/or* hayfever in combination with allergy to furred pets *and/or* pollen for either or both parents at the time of enrolment

**Fig 1:**
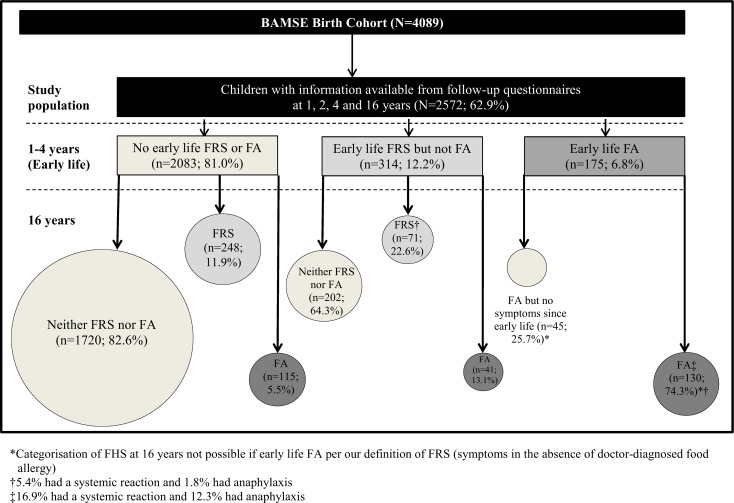
Subjects of the BAMSE birth cohort included in the study population and outcomes of symptoms of food-related symptoms (FRS) and food allergy (FA) at 16 years. *Categorisation of FHS at 16 years not possible if early life FA per our definition of FRS (symptoms in the absence of doctor-diagnosed food allergy). †5.4% had a systemic reaction and 1.8% had anaphylaxis. ‡16.9% had a systemic reaction and 12.3% had anaphylaxis.

Early life FRS and FA prevalence rates were 12.2% and 6.8%, respectively. Of the children with early life FRS, 35.7% also had FRS or FA at 16 years. Yet, early life FRS corresponded to a nearly three-fold increased odds of FRS or FA at 16 years compared to children without early life FRS (OR 2.67; 95%CI 2.05–3.47). In contrast, most (74.3%) children with early life FA also had FA at 16 years, corresponding to a seven-fold increased odds of FA at 16 years (OR 6.94; 95%CI 5.69–8.47). Most children (82.6%) with neither early life FRS nor FA did not have FRS or FA at 16 years (Fig 1).

Amongst children with FRS or FA at 16 years, 5.4% had systemic reactions and 1.8% had anaphylaxis. The corresponding proportions for children with FA at 16 years were 16.9% and 12.3% (both p<0.005 vs. respective proportions for FRS or FA at 16 years).

### Risk factors for FRS and FA in early life to 16 years

Risk factors for FRS or FA at 16 years amongst the 314 children with early life FRS but not FA were parental allergy (OR 2.18; 95%CI 1.32–3.61), early life eczema (OR 2.15; 95%CI 1.32–3.48), rhinitis (OR 2.23; 95%CI 1.26–3.96) and at least one allergic mulitmorbidity (OR 2.03; 95%CI 1.20–3.41) (Table 2 Panel A).

**Table 2:** Risk factors in relation to symptoms of food-related symptoms (FRS) and food allergy (FA) at 16 years amongst children with symptoms of early life FRS or FA, respectively, adjusted analyses*.

	A. Risk factors for FRS or FA at 16 years amongst children with early life FRS				B. Risk factors for FA at 16 years amongst children with early life FA			
	n	N	OR	95%CI	n	N	OR	95%CI
Baseline characteristics								
Sex†								
Female	59	162	1.00		48	72	1.00	
Male	52	147	0.99	0.62–1.60	80	100	**2.13**	**1.05–4.31**
Parental migration status								
Swedish-born	86	236	1.00		92	127	1.00	
Non-Swedish-born	24	67	1.08	0.61–1.94	35	44	1.89	0.80–4.50
Socio-economic status								
Blue collar workers	13	44	1.00		24	31	1.00	
White collar workers	98	265	1.38	0.68–2.79	104	141	0.97	0.37–2.53
Parental allergy‡								
No	66	217	1.00		63	93	1.00	
Yes	45	92	**2.18**	**1.32–3.61**	65	79	**2.33**	**1.11–4.90**
Tobacco smoking in pregnancy								
No	95	268	1.00		120	159	1.00	
Yes	16	41	1.23	0.61–2.47	8	13	0.45	0.13–1.52
Infant feeding								
Exclusive breastfeeding								
< 4 months	22	66	1.00		21	28	1.00	
≥ 4 months	89	243	1.08	0.60–1.93	107	144	0.80	0.30–2.11
Early life allergic mulitmorbidity (to 4 years)								
Asthma								
No	92	259	1.00		89	126	1.00	
Yes	18	48	0.97	0.50–1.87	37	44	1.82	0.72–4.59
Eczema								
No	44	158	1.00		31	48	1.00	
Yes	67	149	**2.15**	**1.32–2.48**	97	124	1.81	0.85–3.86
Rhinitis								
No	76	237	1.00		65	97	1.00	
Yes	34	67	**2.23**	**1.26–3.96**	61	72	**2.30**	**1.02–5.22**
At least one of the above								
None	30	114	1.00		22	35	1.00	
At least one	79	184	**2.03**	**1.20–3.41**	102	132	1.79	0.78–4.09

*Adjusted for sex, parental allergy and socio-economic status

†Adjusted only for parental allergy and socio-economic status

‡Adjusted only for sex and socio-economic status

Bold text denotes statistically significant results

Risk factors for FA at 16 years amongst the 175 children with early life FA included parental allergy (OR 2.33; 95%CI 1.11–4.90) and early life allergic multimorbidity, although the association was statistically significant for early life rhinitis only (OR 2.30; 95%CI 1.02–5.22; Table 2 Panel B). In addition, male sex was associated with an increased risk of FA at 16 years (OR 2.13; 95%CI 1.05–4.31).

### Early life markers of severity and FRS and FA at 16 years

Amongst children with early life FRS but not FA, first reaction by 2 years (87.4%) was more common than a first reaction between 2 and 4 years (12.6%). Children who had a first reaction between 2 and 4 years had a decreased risk of both FRS and FA at 16 years (OR 0.42; 95%CI 0.22–0.81; Table 3 Panel A). Of the early life markers of severity considered, respiratory symptoms (OR 4.65; 95%CI 1.26–17.2) and reactions to peanuts/tree nuts (OR 3.29; 95%CI 1.32–8.16) or fruits with pips or pits (OR 2.97; 95%CI 1.00–8.83), were associated with FRS or FA at 16 years.

**Table 3:** Early life food-related symptoms (FRS) or food allergy (FA) severity as a predictor of FRS or FA at 16 years, adjusted analyses*.

	A. Risk factors for FRS or FA at 16 years amongst children with early life FRS				B. Risk factors for FA at 16 years amongst children with early life FA			
	n	N	OR*	95%CI	n	N	OR*	95%CI
Age at first reaction time								
By 2 years	97	246	1.00		119	158	1.00	
Between 2 and 4 years	14	63	**0.42**	**0.22–0.81**	9	14	0.56	0.17–1.85
Symptoms†								
Gastrointestinal	16	42	1.00		13	22	1.00	
Dermatological‡	44	109	1.22	0.57–2.58	53	65	**2.94**	**1.00–8.67**
Respiratory§	12	16	**4.65**	**1.26–17.2**	35	41	**3.50**	**1.00–12.2**
By specific food†								
Milk	19	66	1.00		21	38	1.00	
Egg	9	24	1.57	0.57–4.30	11	17	1.69	0.50–5.73
Fish	10	32	1.15	0.45–2.94	3	5	0.84	0.12–5.99
Peanuts, tree nuts	18	31	**3.29**	**1.32–8.16**	24	28	**5.63**	**1.60–19.9**
Fruit, pips or pits	11	19	**2.97**	**1.00–8.83**	16	20	3.17	0.87–11.6
Fruit, citrus	17	54	1.10	0.49–2.47	8	11	2.05	0.44–9.45
Chocolate	12	28	1.17	0.67–4.42	18	24	2.27	0.72–7.15

*Adjusted for sex, parental allergy, socio-economic status and early life allergic multimorbidity

†Categories are not mutually exclusive

‡Urticaria *and/or* oedema

§Rhinitis *and/or* wheezing

Bold text denotes statistically significant results

Similarly, more children with early life FA had a first reaction by 2 years (93.0%) than between 2 and 4 years (7.0%). Children who had a first reaction between 2 and 4 years tended to have a reduced risk of FA at 16 years compared to children with a first reaction by 2 years (OR 0.57; 95%I 0.17–1.93; Table 3 Panel B). Other statistically significantly early life markers of severity as a predictor of FA at 16 years included dermatological symptoms (OR 2.94; 95%CI 1.00–8.67) and respiratory symptoms (OR 3.50; 95%CI 1.00–12.2), and reactions to peanuts/tree nuts (OR 5.63; 95%CI 1.60–19.9).

### IgE reactivity as a predictor of FRS and FA to 16 years

Amongst children with early life FRS and for whom IgE data were available, 26.2% (58/221) had IgE reactivity at 4 years (Table 4 Panel A). Early life IgE-associated FRS increased the risk of FRS or FA at 16 years compared to early life non-IgE-associated FRS (OR 3.73; 95%CI 1.93–7.21).

**Table 4:** Children with early life food-related symptoms (FRS) or food allergy (FA), and for whom IgE reactivity data at 4 years were available in relation to FRS or FA at 16 years, adjusted analyses*.

	A. Risk factors for FRS or FA at 16 years amongst children with early life IgE-associated FRS				B Risk factors for FA at 16 years amongst children with early life IgE-associated FA			
	n	N	OR	95%CI	n	N	OR	95%CI
IgE reactivity at 4 years								
No (<0.35 kU_A_/L)	44	163	1.00		26	46	1.00	
Yes (≥0.35 kU_A_/L)	32	58	**3.73**	**1.93–7.21**	68	80	**3.37**	**1.39–8.19**
Allergen-specific IgE reactivity at 4 years								
None	44	163	1.00		26	46	1.00	
Aeroallegens only	13	24	**3.70**	**1.47–9.31**	8	12	1.24	0.30–5.03
Food allergens only	6	16	1.45	0.46–4.56	13	16	3.52	0.83–14.9
Aeroallergens and food allergens	13	18	**9.28**	**2.95–29.1**	47	52	**4.99**	**1.60–15.5**

*Adjusted for sex, parental allergy and socio-economic status

Bold text denotes statistically significant results

To further disentangle the association between IgE reactivity at 4 years and FRS or FA at 16 years, we evaluated IgE by aeroallergens and/or food allergens. Among children with early life FA, 41.4% had IgE reactivity to aeroallergens only, 27.6% to food allergens only, and 31.0% to both aeroallergens and food allergens. Unlike children who had IgE reactivity to aeroallergens only (OR 3.70; 95%CI 1.47–9.31) or both aeroallergens and food allergens (OR 9.28; 95%CI 2.95–29.1), children with IgE reactivity to food allergens only did not have a statistically significantly increased risk of FRS or FA at 16 years.

Amongst children with early life FA and for whom allergen-specific IgE data were available, 54.0% (68/126) had IgE reactivity at 4 years (Table 4 Panel B). IgE reactivity at 4 years was associated with an increased risk of FRS or FA at 16 years compared to no IgE reactivity at 4 years (OR 3.73; 95%CI 1.93–7.21). Of these children, the majority (58.8%) were reactive to both aeroallergens and food allergens at 4 years. Similar to the results for children with early life FRS, children with early life FA and IgE reactivity at 4 years had a statistically significantly increased risk of FA at 16 years (OR 3.37; 95%CI 1.39–8.19). Early life FRS and IgE reactivity to either aeroallergens only or food allergens only at 4 years was not statistically significantly associated with FA at 16 years.

Given that we had allergen-specific IgE data for a subset of the study population at 16 years, we further wanted to examine the association between IgE reactivity at 4 years and food IgE reactivity-associated FRS or FA at 16 years. Amongst the children with early life FRS, 70.4% (19/27) had IgE reactivity already at 4 years (S4 Table, Panel A), of whom 57.9% had food IgE reactivity-associated FRS or FA at 16 years. Amongst the children with early life FA, 92.6% (50/54) had IgE reactivity already at 4 years (S4 Table, Panel B), of whom 86.0% had food IgE reactivity-associated FA at 16 years. Estimations of risk were not possible due to small numbers.

## Discussion

In this large, population-based study of Swedish children followed from birth to 16 years, early life FRS was approximately twice as common as early life FA. The majority of children with early life FA reported FA also at 16 years, whereas most children with early life FRS did not report symptoms at 16 years. Anaphylaxis at 16 years was more common amongst children with early life FA, compared to children with early life FRS. Parental allergy, early life allergic multimorbidity and early life reactions to peanuts or tree nuts and IgE reactivity were common risk factors for FRS or FA at 16 years amongst children with these phenotypes in early life. Amongst children with early life FA only, male sex was associated with an increased risk.

We highlight the strengths of this study. BAMSE is a large, unselected cohort with high retention and detailed follow-up, including types and reactions to foods, through 16 years. To our knowledge, this is the first report on the FRS and FA from early life to adolescence using questionnaire data in combination with sera analyses for IgE reactivity to both aeroallergens and food allergens.

We also acknowledge the limitations of this study. FRS and FA were defined based on parental reports. Further, FA was based on reports of physician diagnosed food allergy. We did not query the type of physician, such as general physician, paediatric allergologist or other, or the type of diagnostic testing or markers of severity, such as levels of mast cell mediators. Thus, we cannot dismiss the possibility of over-reported FA. However, the rates of both early life FRS (12.2%) and FA (6.8%) align with the current global paediatric estimates of 11–22% [2–4] and 4–8% [5–7] for the two phenotypes, respectively. We also lacked data on the frequency of reactions and treatments in early life, and thus cannot state if either has any predictable influence on FRS or FA in adolescence. Other Swedish studies would support that previously known food allergy and previous prescriptions for adrenaline autoinjectors are a risk factor for emergency department revisits for children with a prior emergency department visit resulting from food reactions [33], but that nearly all (89%) of children are hospitalised only once for allergic reactions [20]. As well, we cannot exclude the possibility that FRS and FA at 16 years were more likely to be reported among children with FRS or FA in early life. Yet, such misclassification is not likely to fully explain the higher prevalence of FRS or FA at 16 years among children with early life FHS or FA, particularly because of the 12-year time lapse between assessments. Further, such misclassification would not explain the observed association between early life risk factors and FA or FRS at 16 years. Lastly, we acknowledge that many of the analyses on specific symptoms and foods are based on small numbers, and thus should be interpreted with caution.

Parental allergy was significantly more common amongst children with early life FA compared to early life FRS. This raises the possibility that children whose parents are allergic may be more likely to receive a food allergy diagnosis. However, parental allergy was associated with a two-fold increased risk of FRS and/or FA at age 16 years among both early life phenotypes.

Our finding that males with early life FA had an increased risk of FA at 16 years, compared to females extends the findings from other birth cohorts with follow-up through school-age [26], and parallels findings in which other allergic diseases were considered [34, 35]. Yet, females from adolescence onward are much more likely to experience severe reactions, including anaphylaxis, compared to similar-aged men, an observation which has been attributed to estrogen playing a role in the susceptibility to anaphylaxis [36]. Early life allergic mulitmorbidity was a risk factor for FRS and FA at 16 years, possibly with the exception of asthma among children with early life FRS. Notably, the point estimates for early life asthma were substantially different for FRS at 16 years vs. FA at 16 years.

FRS and FA symptoms frequently involve the gastrointestinal and dermatological systems [8, 37]. In our study, compared to gastrointestinal symptoms, all other symptoms increased the risk of FA at 16 years, while only the most severe symptoms (i.e. respiratory) were associated with an increased risk of FRS at 16 years. Respiratory symptoms are often [10] but not always [38] associated with severe reactions, including anaphylaxis. In our study, anaphylaxis at 16 years was more common in children with FA in both early life and at 16 years, compared to children with early life FRS and FRS or FA at 16 years (12.3% vs. 1.8%), thereby reinforcing that FA is the more severe phenotype.

Like our study, many [39–43] but not all [15, 38, 44, 45] previous studies on FRS or FA were prospective in design. But, only one previous study included follow-up to adolescence [43]. Most focused on allergies to individual foods [39–45], particularly milk [39–41, 45], egg [42, 44] and peanut [38, 43]. Although these foods warrant attention given the commonality of allergy to these foods amongst children [8], restricting analyses to individual foods may not capture a complete picture of the natural history of FRS or FA from early life through adolescence. In our study of seven foods, we found that children with early life reactions to peanuts/tree nuts were more than three times more likely to experience FRS or FA at 16 years than children who reacted to milk in early life. Although we cannot conclude that those with FRS or FA at 16 are still allergic to peanuts/tree nuts, our findings align with previous reports that children typically do not outgrow reactions to peanuts [43]. Interestingly, amongst children with early life FRS only, early life reactions to fruits with pips or pits were also associated with an increased risk of FRS or FA at 16 years. This finding extends previous results from our cohort, in which symptoms to pollen and/or fruit by 2 years of age were associated with an increased risk of allergic diseases, including asthma, rhinitis and eczema [46]. Our observation that children with early life FRS are commonly sensitised to aeroallergens only aligns with findings from other studies [3, 4] and supports the theory that reactions to fruits with pips or pits are often the result of these cross-reactions. In contrast, IgE reactivity to both aeroallergens and food allergens predominated amongst children with either early life FRS or FA. Peanuts and tree nuts are common triggers of anaphylaxis [8], whereas reactions to fruits with pips and pits tend to involve the oral cavity as a result of cross-reactivity to aeroallergens, such as birch [47]. Yet, systemic reactions and anaphylaxis predominated amongst children with early life FA compared to early life FRS, suggesting that early life reactions ought to be considered in combination with a clinical definition when predicting the severity of future reactions.

Despite several common risk factors for persistence into adolescence, early life FRS and FA appear to be different phenotypes with different prognoses though adolescence. Although our findings must be confirmed in other populations, they may also begin to help answer the question that parents frequently ask: Will s/he outgrow it?

In conclusion, food-related symptoms are twice as common as food allergy in early life. Unlike food allergy, food-related symptoms often remit by adolescence. Yet, these phenotypes have many common risk factors for persistence to adolescence.

## Supporting Information

S1 Table Definition of food-induced anaphylaxis at 16 years, based on criteria from the National Institute of Allergy and Infectious Disease/Food Allergy and Anaphylaxis Network. *Based on Reference 32. †Excludes information on blood pressure, heart rate or oxygen saturation, as these data were not collected in the BAMSE study.
(DOCX)

S2 Table Definitions of baseline characteristics, infant feeding, early life allergy multimorbidity and Immunoglobulin E (IgE) activity. (DOCX)

S3 Table Distribution of baseline characteristics and infant feeding of the entire cohort and the study population. *Parent.report of doctor-diagnosis of asthma *and/or* hayfever in combination with allergy to furred pets by either or both parents at time of enrolment. †Mother reporting smoking 1+ cigarettes per day during pregnancy. Bold text denotes statistically significant results.
(DOCX)

S4 Table Children with early life food-related symptoms (FRS) or food allergy (FA) and for whom IgE-reactivity data at 4 years were available in relation to food IgE reactivity-associated FRS or FA to 16 years. (DOCX)

## Abbreviations

FA: Food allergy
FRS: Food-related symptoms
IgE: Immunoglobulin E
OR: Odds ratio
95%CI: 95^th^ per cent confidence intervals
