# Supplementary material for: Food-Related Symptoms and Food Allergy in Swedish Children from Early Life to Adolescence
Source: PLoS One. 2016 Nov 15;11(11):e0166347. doi: 10.1371/journal.pone.0166347 (PMC5112902; doi:10.1371/journal.pone.0166347)
Supplement: S1 Table — *Based on Reference 32. †Excludes information on blood pressure, heart rate or oxygen saturation, as these data were not collected in the BAMSE study. (DOCX) [file pone.0166347.s001.docx]

| **S1 Table.** Definition of food-induced anaphylaxis at 16 years, based on criteria from the National Institute of Allergy and Infectious Disease | |
| --- | --- |
| /Food Allergy and Anaphylaxis Network*† | |
|  | |
| **Anaphylaxis: Involvement of two or more of the following organ systems and symptoms** | |
| Organ system | Symptoms |
| Dermatological | Generalised urticaria |
|  | Facial oedema |
| Lower respiratory | Breathing difficulties |
|  | Asthma |
|  | Dyspnoea |
|  | Cough |
|  | Hoarseness |
|  | Indistinct speech |
| Gastrointestinal | Vomiting |
|  | Stomach ache |
| Cardiovascular | Unconsciousness |
|  |  |
| *Based on Reference 32 |  |
| **†**Excludes information on blood pressure, heart rate or oxygen | |
| saturation, as these data were not collected in the BAMSE study | |
