# Supplementary material for: Food-Related Symptoms and Food Allergy in Swedish Children from Early Life to Adolescence
Source: PLoS One. 2016 Nov 15;11(11):e0166347. doi: 10.1371/journal.pone.0166347 (PMC5112902; doi:10.1371/journal.pone.0166347)
Supplement: S2 Table — (DOCX) [file pone.0166347.s002.docx]

| **S2 Table.** Definitions of baseline characteristics, infant feeding, early life allergic multimorbidity and Immunoglobulin E (IgE) reactivity | |
| --- | --- |
|  |  |
| Variable | Definition |
| Baseline characteristics |  |
| Swedish-born parents | Both parents report being born within Sweden |
| White collar families | Socio-economic status for the household according to Swedish socio-economic classifications; categorised as white |
|  | collar workers *or* blue collar workers |
| Parental allergy | Mother *and/or* father with doctor-diagnosed asthma and asthma medication *and/or* doctor-diagnosed rhinitis in |
|  | combination with furred pets- *and/or* pollen allergy at baseline |
| Infant feeding |  |
| Exclusive breastfeeding | Parent-reported duration of exclusive breastfeeding, dichotimised as < 4 months or ≥ 4 months |
| Early life allergic multimorbidity |  |
| Early life asthma | 3+ episodes of wheeze after 3 months and up to 2 years in combination with treatment with inhaled glucocorticoids |
|  | *and/or* sign of suspected hyperactivity without concurrent upper respiratory infection *and/or* at least 4 episodes of |
|  | wheeze in the past 12 months *or* 1+ episodes of wheeze during the same period combined with a prescription for |
|  | inhaled steroids for symptoms of asthma between 2-4 years |
| Early life eczema | Dry skin and itchy rashes for 2+ weeks with specific localisation of rash *and/or* doctor-diagnosed eczema reported in the |
|  | questionnaires at 1 year *and/or* 2 years *and/or* 4 years |
| Early life rhinitis | Symptoms from eyes/nose (suspected or evident) after exposure to furred pets *and/or* pollen *and/or* |
|  | doctor-diagnosed allergic rhinitis reported in the questionnaires at 1 year and/or 2 years and/or 4 years |
| At least one early life allergic multimorbidity | 1+ reports of early life asthma, eczema *and/or* rhinitis |
|  |  |
| IgE reactivity |  |
| No IgE reactivity | IgE-value for both Phadiatop® *and* fx5® <0.35 kU_A_/L |
| IgE reactivity to aeroallergens only | IgE-value for Phadiatop® ≥ 0.35 kU_A_/l *but* fx5® mix < 0.35 kUA/L |
| IgE reactivity to food allergens only | IgE-value for Phadiatop® < 0.35 kU_A_/l *but* fx5® mix ≥ 0.35 kUA/L |
| IgE reactivity to both aeroallergens and food | IgE-value for Phadiatop® ≥ 0.35 kU_A_/l *and* fx5® ≥ 0.35 kU_A_/L |
