# Supplementary material for: Food-Related Symptoms and Food Allergy in Swedish Children from Early Life to Adolescence
Source: PLoS One. 2016 Nov 15;11(11):e0166347. doi: 10.1371/journal.pone.0166347 (PMC5112902; doi:10.1371/journal.pone.0166347)
Supplement: S3 Table — *Parent.report of doctor-diagnosis of asthma and/or hayfever in combination with allergy to furred pets by either or both parents at time of enrolment. †Mother reporting smoking 1+ cigarettes per day during pregnancy. Bold text denotes statistically significant results. (DOCX) [file pone.0166347.s003.docx]

| **S3 Table .** Distribution of baseline characteristics and infant feeding of the entire cohort and the study population | | | | | | |
| --- | --- | --- | --- | --- | --- | --- |
|  |  |  |  |  |  |  |
|  | **Entire Cohort** | |  | **Study Population** | | |
|  | (n=4089) | |  | (n=2572) | | |
| Baseline characteristics | n | Percent |  | n | Percent | (95% CI) |
| Females | 2024/4089 | 49.5 |  | 1294/2572 | 50.3 | (48.4 – 52.2) |
| Swedish-born parents | 2691/2409 | 78.9 |  | 1990/2482 | 80.2 | (78.6 - 81.7) |
| White collar families | **3323/4018** | **82.7** |  | **2155/2544** | **84.7** | **(83.3 - 86.1)** |
| Parental allergy* | 1200/4041 | 29.7 |  | 765/2549 | 30.0 | (28.2 - 31.9) |
| Tobacco smoking in pregnancy† | **527/4088** | **12.9** |  | **293/2572** | **11.4** | **(10.2 - 12.7)** |
| Infant feeding |  |  |  |  |  |  |
| Exclusive breastfeeding for ≥ 4 months | 3116/3919 | 79.5 |  | 2078/2570 | 80.9 | (79.3 - 82.4) |
| *Parent-report of doctor-diagnosis of asthma *and/or* hayfever in combination with allergy to furred | | | | | | |
| pets by either or both parents at time of enrollment | |  |  |  |  |  |
| †Mother reported smoking 1+ cigarettes per day during pregnancy | | | | | |  |

Bold text denotes statistically significant results
