# Supplementary material for: Food-Related Symptoms and Food Allergy in Swedish Children from Early Life to Adolescence
Source: PLoS One. 2016 Nov 15;11(11):e0166347. doi: 10.1371/journal.pone.0166347 (PMC5112902; doi:10.1371/journal.pone.0166347)
Supplement: S4 Table — (DOCX) [file pone.0166347.s004.docx]

| **S4 Table**. Children with early life food-related symptoms (FRS) or food allergy (FA) and for whom IgE-  reactivity data at 4 years were available in relation to food IgE reactivity-associated FRS or FA to 16 years | | | | | | | |
| --- | --- | --- | --- | --- | --- | --- | --- |
|  |  |  |  |  |  |  |  |
|  | A. Early life FRS and food IgE reactivity-associated FRS or FA at 16 years | | |  | B. Early life FA and food IgE reactivity-associated FA at 16 years | | |
|  |  |  |  |  |  |  |  |
|  | n | N | % |  | n | N | % |
| IgE reactivity at 4 years |  |  |  |  |  |  |  |
| No ( <0.35 kU_A_/L ) | 0 | 8 | 0.0 |  | 4 | 4 | 100.0 |
| Yes ( ≥0.35 kU_A_/L) | 11 | 19 | 57.9 |  | 43 | 50 | 86.0 |
| Allergen-specific IgE reactivity at 4 years |  |  |  |  |  |  |  |
| None | 0 | 8 | 0.0 |  | 4 | 4 | 100.0 |
| Aeroallegens only | 2 | 6 | 33.3 |  | 1 | 2 | 50.0 |
| Food allergens only | 2 | 4 | 50.0 |  | 5 | 8 | 62.5 |
| Aeroallergens and food allergens | 7 | 9 | 77.8 |  | 37 | 40 | 92.5 |
